# Retraction: Optimizing sgRNA to improve CRISPR/Cas9 knockout efficiency: special focus on human and animal cell

**DOI:** 10.3389/fbioe.2023.1285036

**Published:** 2023-09-04

**Authors:** Frontiers Editorial Office

Following publication, concerns were raised regarding the contributions of the authors of the article.

Our investigation, conducted in accordance with Frontiers policies, confirmed a serious breach of our authorship policies and of publication ethics; the article is therefore retracted.

The authors do not agree to this retraction.

This retraction was approved by the Chief Editors of Frontiers in Bioengineering and Biotechnology and the Chief Executive Editor of Frontiers.

